# A Case Report of Choroidal Neovascularization Secondary to Angioid Streaks Associated With Beta Thalassemia

**DOI:** 10.7759/cureus.84800

**Published:** 2025-05-25

**Authors:** Thanam Tamil Chelvan, Teck Chee Cheng, Jemaima Che Hamzah, Ainal Adlin Naffi

**Affiliations:** 1 Department of Ophthalmology, Hospital Canselor Tuanku Muhriz, Universiti Kebangsaan Malaysia, Kuala Lumpur, MYS

**Keywords:** angioid streaks, anti-vascular endothelial growth factor, beta thalassemia, choroidal neovascularization, optical coherence tomography macula

## Abstract

A 58-year-old Malay male with underlying beta thalassemia presented with distorted vision in the right eye for two weeks. On presentation, his visual acuity (VA) was 6/18 OD (right eye) and 6/9 OS (left eye). Fundus examination of the right eye revealed a pink optic disc with peripapillary atrophy and numerous narrow, irregular streaks radiating in a circumferential pattern, along with macular scarring and pigment deposition. The patient was ametropic, with a right eye axial length of 24.72 mm.

Optical coherence tomography (OCT) of the macula of the right eye showed subretinal and intraretinal cystic fluid with subretinal fibrosis. Fundus fluorescein angiography demonstrated leakage at 44 seconds, increasing in intensity and size in the early venous phase. The patient was diagnosed with choroidal neovascularization (CNV) secondary to angioid streaks. He was treated with intravitreal ranibizumab, receiving a monthly loading dose for three months, followed by a treat-and-extend regimen up to 16-week intervals. The patient’s symptoms resolved after the first injection, and follow-up OCT showed sustained resolution of fluid. Final VA remained 6/18 OD and 6/9 OS, with symptomatic improvement and stability of the condition.

## Introduction

Beta thalassemia is an autosomal recessive blood disorder caused by a mutation in the gene encoding for the beta chains of hemoglobin. It is among the most common hemoglobinopathies. Individuals with beta thalassemia major require regular blood transfusions to survive and to prolong their lives; however, regular blood transfusion leads to iron depositions in the tissue and may eventually result in multi-organ dysfunction, including the eye. In the eye, abnormal iron homeostasis in hereditary diseases can lead to iron overload and degenerative changes in the retina [1,2].

Ocular pathology, such as ocular surface disorders, cataract, angioid streak, retinal venous tortuosity, retinal toxicity, retinal pigment epithelium (RPE) degeneration and mottling, optic neuropathy, and decreased visual acuity, has been reported to be associated with beta thalassemia. Angioid streaks have been documented in various hemoglobinopathies, underscoring a significant association between these systemic disorders and degenerative changes in Bruch’s membrane [1,3].

Angioid streaks and consecutive choroidal neovascularization (CNV) are the least frequently reported causes of loss of visual acuity in beta thalassemia. These ocular changes can occur as a result of the disease itself, massive iron deposition, or the side effects of the iron chelators [1,3].

This article was previously presented as an e-poster at the 13th Conjoint Ophthalmology Scientific Conference (COSC 2024), held from 13th to 14th September 2024 in Kota Bharu, Kelantan.

## Case presentation

A 58-year-old Malay gentleman with underlying beta thalassemia major and benign prostate hypertrophy presented with right eye distorted vision centrally for two weeks. The distortion was persistent and not worsening over time. There were no previous episodes of similar complaints. He denied any micropsia or macropsia prior to the presentation. He presented to our clinic two weeks after the onset due to no visual recovery.

Upon presentation, his visual acuity (VA) was 6/18 OD (right eye) and 6/9 OS (left eye), with a near vision of N18 OD and N6 OS. The anterior segment findings for both eyes were unremarkable. The intraocular pressure (IOP) was 14 mmHg in both eyes.

**Figure 1:**
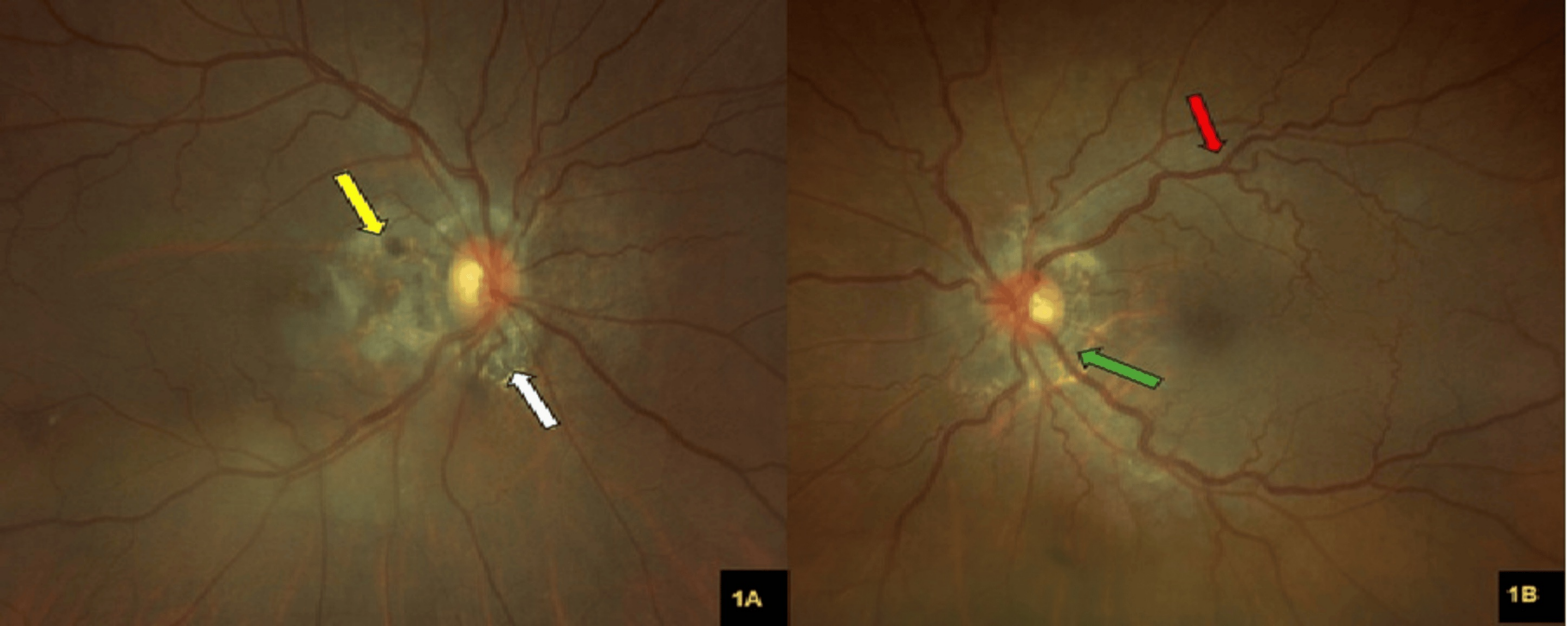
(A) A colored fundus photograph of the right eye shows a pink optic disc with angioid streak (white arrow), along with macula scarring and some greyish pigment deposition (yellow arrow). (B) A colored fundus photograph of the left eye shows a pink optic disc with a peripapillary ring of atrophy with numerous narrow, irregular streaks radiating in a circumferential pattern (green arrow). The retinal vessels appear slightly tortuous and dilated (red arrow).

The right fundus revealed a pink optic disc with a peripapillary ring of atrophy with numerous narrow irregular streaks radiating in a circumferential pattern, along with macula scarring and some greyish pigment deposition (Figure 1A). Otherwise, the optic disc was pink with a cup-to-disc ratio (CDR) of 0.4. There were no retinal hemorrhages or drusen seen. The left eye fundus showed a pink optic disc with a CDR of 0.4, with a normal macula. No retinal hemorrhages were seen (Figure 1B).

**Figure 2:**
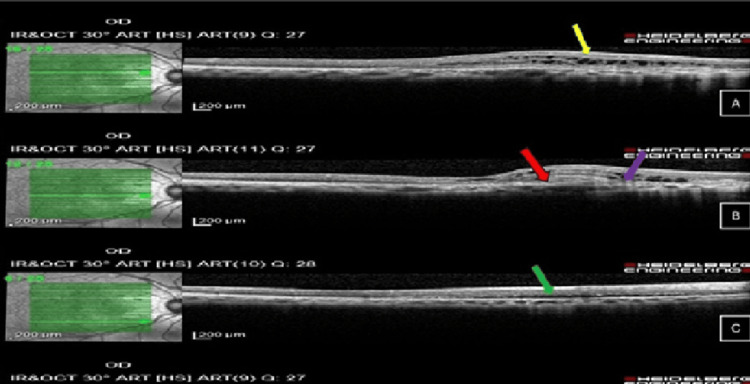
Optical coherence tomography of the right eye macula shows (A) cystic intraretinal fluid (yellow arrow), (B) subretinal fibrosis (red arrow) and disrupted ellipsoid zone (purple arrow), with (C) subretinal fluid (green arrow).

Optical coherence tomography (OCT) of the right eye macula showed cystic intraretinal fluid and subretinal fluid with subretinal fibrosis, as shown in Figure 2 at different levels.

**Figure 3:**
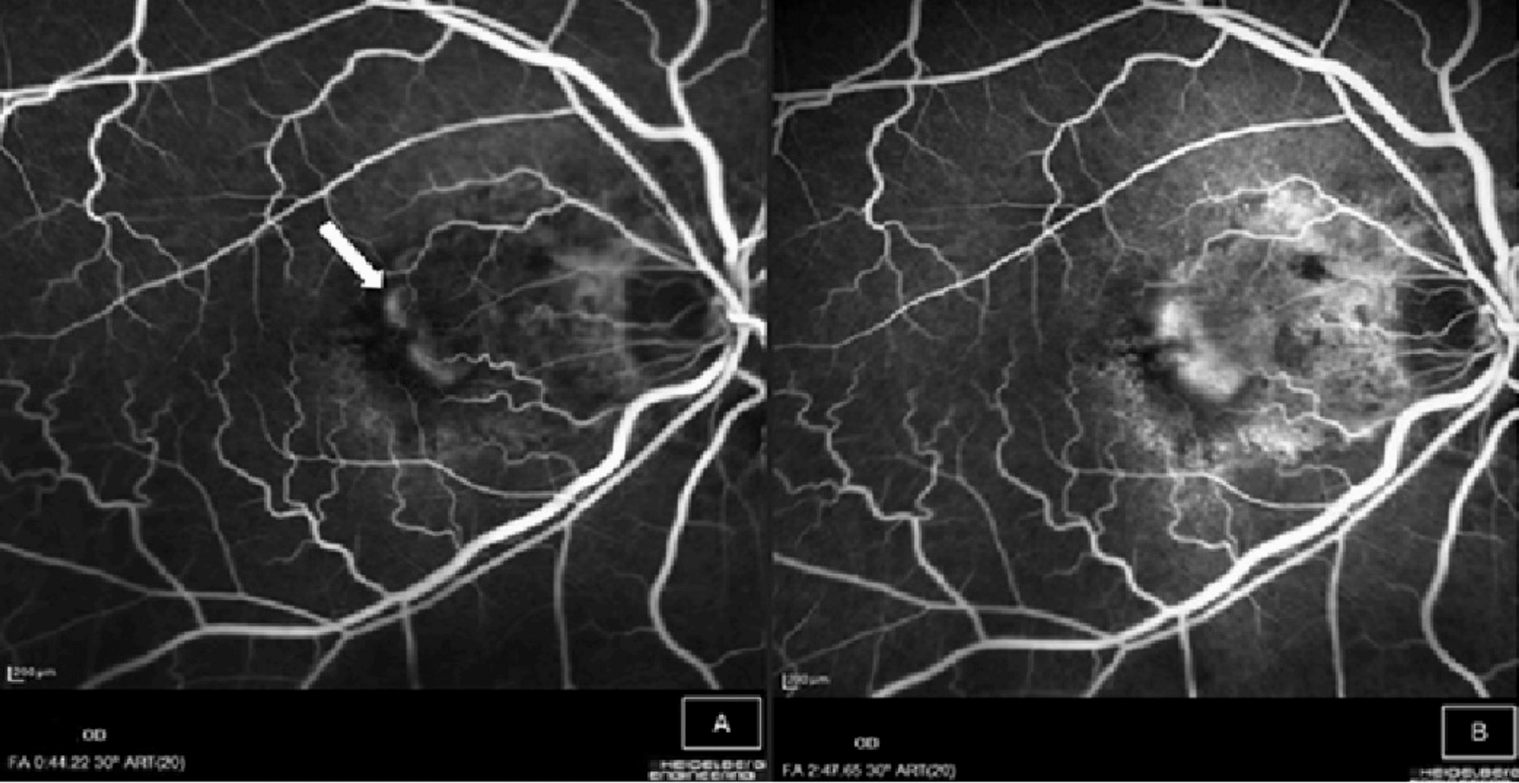
Fundus fluorescein angiography of the right eye shows (A) hyperfluorescence (white arrow) at venous phase (44 seconds), which increases in size and intensity until two minutes and 47 seconds (B).

Fundus fluorescein angiography was performed, and it showed leakage at 44 seconds in the early venous phase, evidenced by increasing size and intensity of the hyperfluorescence, suggesting CNV (Figure 3).

**Figure 4:**
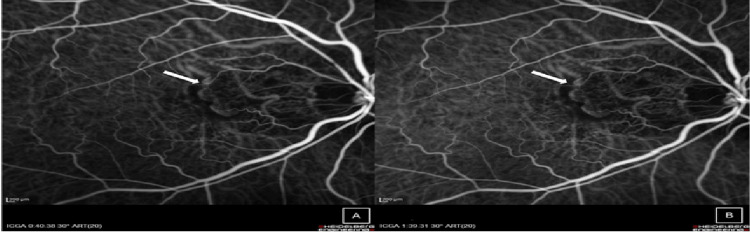
Indocyanine green angiography revealed a well-demarcated area of hypofluorescence at the macula (white arrow), corresponding to the site of suspected choroidal neovascularization. The lesion remained hypofluorescent from the mid phase (A, 40 seconds) to the late phase (B, 99 seconds).

On indocyanine green angiography, a clearly demarcated hypofluorescent area was observed at the macula, correlating with the suspected choroidal neovascular membrane. The sustained hypofluorescence between the mid phase (40 seconds) and late phase (99 seconds) suggests underlying subretinal fibrosis (Figure 4).

The patient was diagnosed with CNV secondary to angioid streaks associated with underlying beta thalassemia. Fundus examination revealed bilateral, narrow, irregular lines deep to the retina, radiating from the optic disc, characteristic of angioid streaks. The diagnosis of CNV was further supported by multimodal imaging findings.

**Figure 5:**
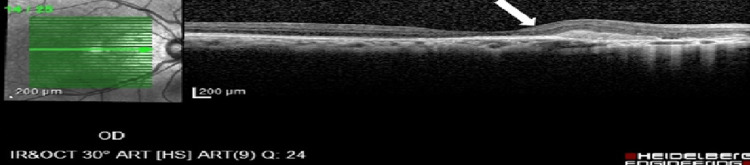
Optical coherence tomography of the right eye showing complete resolution of the intraretinal and subretinal fluid with subretinal fibrosis (white arrow).

This patient was managed with intravitreal anti-vascular endothelial growth factor (anti-VEGF), namely, ranibizumab, injection over the right eye, with a monthly loading dose for three months, followed by a treat-and-extend regimen up to 16 weeks. His symptoms resolved after the first injection, and OCT of the macula showed resolution of intraretinal and subretinal fluid after one month post first intravitreal injection (Figure 5).

His final VA was 6/18 OD and 6/9 OS, with improvement of the symptoms, and there was no recurrence up to date.

## Discussion

Patients with beta thalassemia major often suffer from profound anemia, complications related to repeated blood transfusions, and iron overload. Retinal pathologies associated with beta thalassemia, as cited in the literature, include RPE degeneration, RPE mottling, angioid streaks, retinal vessel tortuosity, retinal hemorrhages, retinal edema, pseudo-papillitis, and macular scarring [2].

Angioid streaks have been reported in various hemoglobinopathies, suggesting a possible association with beta thalassemia as well. However, the pathogenesis of angioid streaks in beta thalassemia remains unclear. It has been postulated that chronic hemolysis results in deposition of iron in Bruch's membrane, which may compromise the integrity of the Bruch’s membrane, creating cracks that radiate out from the optic disc. These cracks allow small blood vessels to ascend into the retinal layer [4,5].

Barteselli et al., in a cross-sectional, observational study, reported that patients with long-standing disease requiring iron-chelating treatment need regular ophthalmic checkups because they are at risk of developing CNV, as iron chelators have ocular effects, such as macular and equatorial pigmentary degeneration and toxicity, mainly affecting the RPE-Bruch membrane-photoreceptor complex [6-8].

In our case, the patient receives blood transfusions every three months. He has an elevated serum ferritin level of 450 µg/L, with moderate hepatic iron overload evidenced by magnetic resonance imaging performed in June 2022 (stage 2: moderate iron overload). He is also on iron chelation therapy. All these factors predispose the patient to the risk of developing CNV. There is peripapillary atrophy with angioid streaks around the optic disc, and the CNV was detected temporal to the optic disc, correlating to the weak area, sparing the fovea region. His symptoms also correlate with the area of CNV, and the patient responded well to anti-VEGF treatment, seen after the first injection. His metamorphopsia completely resolved, even though there is no improvement in visual acuity due to macula scarring.

This report indicates the efficacy of anti-VEGF treatment in a patient with concurrent beta thalassemia and CNV, consistent with previous reports that considered anti-VEGF as a safe and effective treatment option for CNVs due to angioid streaks [9]. The treatment of this condition remains a therapeutic challenge, as it has a high rate of recurrence and natural progression of fibrosis and atrophic changes. There is no general consensus about the most appropriate treatment or dosing strategy (PRN (pro re nata), treat and extend, or fixed); however, anti-VEGF intravitreal injections were found to be effective. Ladas et al. reported that the treat-and-extend regimen is highly effective in treating CNV secondary to angioid streak and in stabilizing the lesion [10]. We opted for a treat-and-extend regimen for better visual and anatomical results, keeping in mind that the patient is under a high-risk category, with moderate iron overload and on iron chelators. There is no recurrence of CNV at one year of follow up and his condition is stable at 16 weeks of interval of anti-VEGF treatment. To our best knowledge, this is the first report on CNV secondary to angioid streak with underlying beta thalassemia major that underwent a treat-and-extend regimen and showed good response to anti-VEGF treatment.

## Conclusions

CNV secondary to angioid streaks is a rare but visually significant complication in patients with beta thalassemia. This case highlights the importance of early recognition and prompt treatment with intravitreal anti-VEGF therapy, which can lead to stabilization of the lesion and resolution of symptoms. The treat-and-extend regimen proved to be an effective approach in this patient, with sustained anatomical and symptomatic improvement and no recurrence up to one year of follow-up. Regular ophthalmic monitoring is essential in beta thalassemia patients, especially those with iron overload and on iron-chelating therapy, as early detection of ocular complications can significantly impact visual outcomes.
